# Editorial to “Carbon dioxide insufflation to facilitate epicardial access in ECMO‐supported ventricular tachycardia ablation”

**DOI:** 10.1002/joa3.13200

**Published:** 2024-12-18

**Authors:** Wen‐Han Cheng, Fa‐Po Chung

**Affiliations:** ^1^ Heart Rhythm Center and Division of Cardiology, Department of Medicine Taipei Veterans General Hospital Taipei Taiwan; ^2^ Department of Medicine, School of Medicine National Yang Ming Chiao Tung University Taipei Taiwan; ^3^ Division of Cardiology, Department of Medicine National Yang Ming Chiao Tung University Hospital Yilan Taiwan

**Keywords:** Carbon dioxide insufflation, epicardial ablation, ventricular tachycardia

In this issue of the Journal, Takase et al. present a compelling case involving a patient with myocardial disease secondary to scleroderma. The patient underwent a repeat epicardial catheter ablation for recurrent VT after the failure of initial endocardial ablation. The authors employed carbon dioxide (CO_2_) insufflation to expand the pericardial space under extracorporeal membrane oxygenation (ECMO) support. This approach mitigated the technical challenges typically associated with epicardial access, particularly in patients with minimal pericardial fluid. The technique demonstrates a significant leap in safety and efficacy, addressing the risks of traditional epicardial access methods.^1^

Catheter ablation has emerged as an alternative treatment option for patients suffering from sustained, monomorphic ventricular tachycardia (VT). Traditional endocardial ablation techniques, leveraging electrophysiological or substrate‐based mapping, have shown promise in reducing the burden of ventricular arrhythmia, achieving acute success rates of 60%–80%.^2^ However, the complex pathology and 3‐dimentional architecture of VT isthmus, often involving diffuse myocardial regions, including the epicardium, poses significant challenges to the long‐term success of these procedures. This has driven interest in epicardial approaches, which are particularly relevant for nonischemic cardiomyopathy‐associated ventricular arrhythmias, where endocardial ablation alone may be insufficient. Recent advancements in endo‐epicardial ablation strategies have demonstrated their potential to enhance outcomes, especially in patients with extensive myocardial involvement. Epicardial ablation becomes crucial in cases when endocardial‐only approaches fail to achieve clinical success. The introduction of innovative techniques for epicardial access has further expanded the possibilities of safe and effective VT management.^2^

The epicardial approach, initially introduced by Sosa et al., involved the use of a nonsurgical transthoracic 18‐G needle for pericardial space access.^3^ Over the years, several innovations have refined this technique to enhance safety and success rates. Methods such as needle‐in‐needle systems, CO_2_ insufflation, real‐time pressure monitoring, blunt‐tip concealed needle devices, video‐assisted approaches, and the SAFER (Safe Access for Epicardial Radiofrequency) technique have reduced complications and improved procedural outcomes.^4^

Among these innovations, the use of CO_2_ insufflation is noteworthy. Initially, intentional CO_2_ insufflation was performed via the right atrial appendage exit. Though previously described, it has not been widely adopted in clinical practice. Later, CO_2_ insufflation via coronary venous system has been introduced to offer a safer, more efficient means of accessing epicardial space by creating a visible cavity on fluoroscopy. This enhanced visualization significantly reduces the risk of complications, such as inadvertent puncture of critical structures like the right ventricle, coronary arteries, or liver.^5^

Takase et al. discuss the benefits of CO_2_ insufflation, highlighting how inflating the pericardial space with CO_2_ creates a clear puncture route for epicardial access.^1^ This technique, which has a reduced learning curve, is more accessible to medical centers with less experience in advanced electrophysiological procedures.^1^ Intentionally perforating coronary vein branches using fluoroscopic imaging was a key innovation. This precision helps avoid complications like damage to nearby structures. The authors also emphasize the importance of CTO wires in safely navigating the epicardial space.

The article also highlights that CO_2_ insufflation may reduce risks of epicardial access in high‐risk patients. The patient's hepatic left lobe posed a risk during the subxiphoid puncture, but the CO_2_ technique created sufficient space by moving the diaphragm downward, eliminating the need for breath‐holding. This method simplifies the procedure and improves patient comfort and safety. Moreover, there was no decrease in blood pressure during the tamponade of the heart with CO_2_ further validates the safety of this technique. Stable blood pressure and no bleeding at the venous exit site highlight CO_2_ insufflation's effectiveness in ensuring safe epicardial access.^1^

In conclusion, the article discusses a safer method for catheter ablation of VT using CO_2_ insufflation to expand the pericardial space in high‐risk patients. This technique mitigates the risks of epicardial access and offers an effective alternative to traditional methods.

## CONFLICT OF INTEREST STATEMENT

Authors declare no conflict of interests for this article.
